# A Non-invasive Model for Predicting Liver Inflammation in Chronic Hepatitis B Patients With Normal Serum Alanine Aminotransferase Levels

**DOI:** 10.3389/fmed.2021.688091

**Published:** 2021-06-04

**Authors:** Xiaoke Li, Yufeng Xing, Daqiao Zhou, Huanming Xiao, Zhenhua Zhou, Zhiyi Han, Xuehua Sun, Shuo Li, Ludan Zhang, Zhiguo Li, Peng Zhang, Jiaxin Zhang, Ningyi Zhang, Xu Cao, Xiaobin Zao, Hongbo Du, Guangdong Tong, Xiaoling Chi, Yueqiu Gao, Yong'an Ye

**Affiliations:** ^1^Department of Gastroenterology, Dongzhimen Hospital, Beijing University of Chinese Medicine, Beijing, China; ^2^Institute of Liver Diseases, Beijing University of Chinese Medicine, Beijing, China; ^3^Department of Hepatology, Shenzhen Traditional Chinese Medicine Hospital, Shenzhen, China; ^4^Department of Hepatology, Guangdong Provincial Hospital of Chinese Medicine, Guangzhou, China; ^5^Department of Hepatopathy, Shuguang Hospital Affiliated to Shanghai University of Traditional Chinese Medicine, Shanghai, China; ^6^Department of Gastroenterology, Beijing Fengtai Hospital of Integrated Traditional and Western Medicine, Beijing, China; ^7^Department of Gastroenterology and Hepatology, Dongfang Hospital Affiliated to Beijing University of Chinese Medicine, Beijing, China

**Keywords:** non-invasive, predictive model, chronic hepatitis B, inflammation, alanine aminotransferase

## Abstract

**Background and Aims:** Chronic hepatitis B (CHB) patients with normal alanine aminotransferase (ALT) levels are at risk of disease progression. Currently, liver biopsy is suggested to identify this population. We aimed to establish a non-invasive diagnostic model to identify patients with significant liver inflammation.

**Method:** A total of 504 CHB patients who had undergone liver biopsy with normal ALT levels were randomized into a training set (*n* = 310) and a validation set (*n* = 194). Independent variables were analyzed by stepwise logistic regression analysis. After the predictive model for diagnosing significant inflammation (Scheuer's system, G ≥ 2) was established, a nomogram was generated. Discrimination and calibration aspects of the model were measured using the area under the receiver operating characteristic curve (AUC) and assessment of a calibration curve. Clinical significance was evaluated by decision curve analysis (DCA).

**Result:** The model was composed of 4 variables: aspartate aminotransferase (AST) levels, γ-glutamyl transpeptidase (GGT) levels, hepatitis B surface antigen (HBsAg) levels, and platelet (PLT) counts. Good discrimination and calibration of the model were observed in the training and validation sets (AUC = 0.87 and 0.86, respectively). The best cutoff point for the model was 0.12, where the specificity was 83.43%, the sensitivity was 77.42%, and the positive likelihood and negative likelihood ratios were 4.67 and 0.27, respectively. The model's predictive capability was superior to that of each single indicator.

**Conclusion:** This study provides a non-invasive approach for predicting significant liver inflammation in CHB patients with normal ALT. Nomograms may help to identify target patients to allow timely initiation of antiviral treatment.

## Introduction

Hepatitis B virus (HBV) infection is a global public health problem and a major contributor to liver cirrhosis and liver cancer. The prevalence of chronic HBV infection varies geographically from 2 to 8%, and there are ~240,000,000 chronic HBV surface antigen (HBsAg) carriers globally (1). China has the largest chronic hepatitis B (CHB) population in the world. The HBsAg-positive rate was estimated to be 7.18% among Chinese individuals aged 1–59 (1–3).

Aggressive antiviral treatment is essential to disrupt the progression of the disease, the initiation of which depends on whether the levels of serum liver enzymes, especially alanine aminotransferase (ALT), are abnormal. Accordingly, various guidelines use an abnormal ALT level as a significant indicator to assess disease progression in CHB patients and to guide the initiation of antiviral therapy. However, ALT levels can fluctuate with time, and single measurements do not indicate the disease stage.

In the natural history of HBV, the phases of “chronic infection” and “chronic hepatitis” are distinguished by ALT. Chronic infection includes the old terminology of “immune tolerant” which is characterized by the presence of serum HBeAg, very high levels of HBV DNA and ALT persistently within the normal range, “inactive carrier” which is characterized by the presence of serum antibodies to HBeAg, undetectable or low HBV DNA levels and normal ALT. The chronic hepatitis (antiviral treatment should be administered in this phase) is characterized by elevated ALT and detectable HBV DNA (4). But in practice, “immune tolerant” and “HBeAg positive chronic hepatitis” are often misclassified since liver inflammation cannot be diagnosed solely on ALT levels.

There is evidence that a proportion (~20%) of the normal ALT population has noticeable liver inflammation, which implies the limitation of ALT levels in predicting the chronic severity of hepatic injury (5–7). It has been reported that the T-cell profile of immune-tolerant CHB is similar to that of immune-active CHB (8). A high level of HBV DNA integration and clonal hepatocyte amplification in immune-tolerant CHB patients indicate that liver cancer may occur even in the early stage of infection (9). Thus, if only serum ALT levels are used to determine whether to initiate antiviral therapy, a considerable number of patients will probably lose the opportunity to receive therapeutic interventions. Hence, more aggressive liver histology assessment is recommended by the Chinese Society of Hepatology, Asian Pacific Association for the Study of the Liver (APASL), European Association for the Study of the Liver (EASL), and AASLD guidelines for those with normal or mildly abnormal ALT levels (4, 10–12).

Liver biopsy remains the most common approach for the assessment of liver inflammation and fibrosis and is the ultimate evaluation method to determine whether to start treatment. According to the latest Chinese guidelines, if a patient is HBV DNA (+) but has normal ALT levels, antiviral therapy should be initiated, provided that evidence shows significant inflammation or fibrosis progression (Scheuer's score ≥ G2 or S2) (12). However, populations with normal ALT levels are often the most reluctant to undergo liver biopsy, and the value of biopsy is limited by sampling error, invasiveness, cost, poor compliance, and contraindications (13). Accordingly, several non-invasive methods for predicting liver fibrosis have been proposed over the past decades, including transient elastography (TE) and FibroTest, and measurement of parameters such as the aspartate aminotransferase-to-platelet ratio index (APRI), γ-glutamyl transpeptidase-to-platelet ratio (GPR), and FIB-4 index (14–17). The APRI and FIB-4 indexes sometimes have poor sensitivity and accuracy for predict CHB-associated fibrosis (18, 19), TE is unable to determine the grade of liver inflammation, and is inaccurate in the presence of inflammatory activity (20, 21).

Assessment of liver inflammation is essential before fibrosis occurs. Reliable diagnostic tools that can diagnose early inflammation, especially in populations with persistent normal serum liver enzyme levels, are urgently needed. To our knowledge, few predictive models for diagnosing liver inflammation in the target population exist. In this study, we included multicenter clinical data from 2013 to 2015 gathered in China to establish a non-invasive diagnostic model to assess liver inflammation in people with normal ALT, AST, and γ-glutamyl transpeptidase (GGT) levels. The model was validated in a separate data set. We also explored whether use of the lower upper limit of normal (ULN) of ALT level, which was suggested by AASLD guideline (35 IU/L in males and 25 IU/L in females, that are lower than the conventional threshold of 40 IU/L), can improve the detection of hepatocyte inflammation.

## Methods

### Study Population

The records of patients with chronic HBV infection who underwent liver biopsy between 2013/01/01 and 2015/12/31 at 4 medical centers (Dongzhimen Hospital, Beijing University of Chinese Medicine, Beijing, China; Shenzhen Traditional Chinese Medicine Hospital, Shenzhen, China; Guangdong Provincial Hospital of Chinese Medicine, Guangzhou, China; and Shuguang Hospital Affiliated to Shanghai University of Traditional Chinese Medicine, Shanghai, China) were inspected. Patients who met the inclusion criteria and none of the exclusion criteria were enrolled.

### Inclusion and Exclusion Criteria

The inclusion criteria of this study were as follows: (1) HBsAg (+) over 6 months; (2) normal serum ALT, AST, and GGT levels (the upper limits of normal were 40, 40, and 50 IU/L, respectively); (3) liver biopsy conducted within ±30 days of serum collection; and (4) no treatment with nucleos(t)ide analogs. We excluded patients (1) with hepatitis C virus, hepatitis D virus, and HIV coinfection; (2) with primary biliary cholangitis and autoimmune hepatitis; (3) with a history of excessive alcohol consumption (20 g per day for women and 40 g for men); (4) with confirmed liver cirrhosis or carcinoma; and (5) who were pregnant.

### Laboratory Assays

Serum HBsAg levels were quantified with an Architect i2000 assay (Abbott Laboratories, Kennett Square, PA, USA), with a dynamic range of 0.05–250 IU/mL. Samples with HBsAg levels higher than 250 IU/mL were diluted and retested. Serum HBV DNA levels were quantified using the COBAS TaqMan assay (Roche Diagnostics, Branchburg, NJ, USA), with the lowest detection limit at 20 IU/mL. All blood samples for quantitative HBsAg and HBV DNA analysis were tested by a clinical examination service provider (Shanghai ADICON Clinical Laboratories, Inc., CAP certification, Shanghai, China).

### Histological Examination

All enrolled patients were subjected to ultrasound-guided percutaneous liver biopsy at least 1.5 cm in length containing more than 7 portal areas with 16-G biopsy needles. The specimens were fixed in formalin, embedded in paraffin, and subjected to hematoxylin-eosin and reticular fiber staining. Two experienced pathologists from each Medical Center who were blinded to the clinical information of the subjects assessed the collected samples. Semiquantitative assessment of liver inflammation was performed using Scheuer's system. An inflammation score G ≥ 2 was defined as the target prediction outcome in the study.

### Statistical Analysis

We did not impute missing data. Categorical variables are shown as numbers and percentages. Continuous variables are shown as the median and interquartile range. Student's *t*-test and the Mann–Whitney non-parametric *U*-test were used to compare continuous variables between two groups.

In the latest AASLD guideline, the suggested upper limit of normal (ULN) for ALT was revised to 35 IU/L for men and 25 IU/L for women (10), whereas other guidelines (e.g., the EASL and Chinese guidelines) currently retain an upper limit of 40 IU/L (4, 12). To explore whether lower ULN brings benefits in diagnosing liver inflammation, we established a new variable named “high-normal ALT” (a binary variable), which was defined as serum ALT between 35–40 IU/L in males and 25–40 IU/mL in females.

The significance of each variable in the training set was assessed by univariate logistic regression analysis to identify independent risk factors for significant liver fibrosis. Variables with *P* < 0.2 by univariate analysis were included in the subsequent multivariate analysis. A multivariate logistic regression using automated variable selection and Akaike's information criterion was applied to select the best predictors for predictive model construction. We used the variance inflation factor for the collinearity diagnosis of the multivariate logistic regression.

A nomogram was formulated based on the results of multivariate logistic regression analysis and by using the RMS package of R. The nomogram is based on proportionally converting each regression coefficient in a multivariate logistic regression on a 0- to 100-point scale. The effect of the variable with the highest β coefficient (absolute value) is assigned 100 points. The points are added across independent variables to derive total points, which are converted into predicted probabilities. The predictive performance of the nomogram was measured by the area under the receiver operating characteristic curve (AUC) and calibration (intercept and slope of the calibration line) with 1 000 bootstrap samples to decrease the overfit bias. Decision curve analyses (DCAs) and plots of net benefit against threshold probability were carried out to evaluate these predictive models by examining the theoretical relation between the threshold probability of developing an event and the relative value of false-positive and false-negative results. The model was re-evaluated in a separate validation set.

In all analyses, *P* < 0.05 indicated significance. Analyses were performed with R software version 4.0.2.

## Results

### Patient Characteristics

We obtained medical records for 504 patients that met all inclusion criteria and no exclusion criteria of the current study. These records were divided into a training set (*n* = 310) and a validation set (*n* = 194) using a randomization sample function in R software. Significant inflammation was observed in 12.7% (64/504) of patients. No significant difference in population characteristics was observed between the patients whose data was included in the 2 datasets (Table 1). The data sets are available from the corresponding author (yeyongan@vip.163.com) upon request.

**Table 1:** Characteristics of the study population.

**Variables**	**All**	**Training set**	**Validation set**	***P*-value**
Population	504	310	194	
Age (years)	38 (32–44)	37 (32–44)	38 (32–45)	0.684
Gender				0.477
Male (%)	294 (58.33)	177 (57.10)	117 (60.31)	
Female (%)	210 (41.67)	133 (42.90)	77 (39.69)	
High-normal ALT				0.476
No (%)	381 (75.60)	231 (74.52)	150 (77.32)	
Yes (%)	123 (24.40)	79 (25.48)	44 (22.68)	
HBeAg status				0.082
Negative (%)	179 (35.52)	101 (32.58)	78 (40.21)	
Positive (%)	325 (64.48)	209 (67.42)	116 (59.79)	
Significant fibrosis				0.716
S < 2 (%)	376 (74.60)	233 (75.16)	143 (73.71)	
S ≥ 2 (%)	128 (25.40)	77 (24.84)	51 (26.29)	
Significant inflammation			0.080	
G < 2 (%)	440 (87.30)	277 (89.35)	163 (84.02)	
G ≥ 2 (%)	64 (12.70)	33 (10.65)	31 (15.98)	
ALT (IU/L)	25.00 (18.00–31.25)	25.00 (18.00–32.00)	24.00 (17.6–31.00)	0.165
AST (IU/L)	22.00 (18.70–26.00)	22.00 (18.50–26.00)	22.90 (19.00–28.00)	0.362
HBsAg (Log10 IU/mL)	3.81 (3.06–4.51)	3.79 (3.04–4.50)	3.85 (3.35–4.51)	0.122
HBeAg (S/CO)	291.37 (0.21–1376.15)	364.52 (0.27–1379.85)	95.82 (0.15–1369.84)	0.170
HBcAb (S/CO)	8.88 (0.01–11.41)	9.09 (0.01–11.43)	8.65 (0.01–11.17)	0.348
HBV DNA (Log10 IU/mL)	7.39 (3.90–8.28)	7.43 (4.01–8.29)	7.2 (3.42–8.23)	0.239
WBC (10E9/L)	5.60 (4.67–6.66)	5.56 (4.64–6.67)	5.62 (4.69–6.65)	0.977
RBC (10E12/L)	4.67 (4.30–5.04)	4.68 (4.32–5.04)	4.67 (4.26–5.05)	0.869
PLT (10E9/L)	192.00 (158.25–227.00)	190.00 (158–227)	192.50 (159.75–228.25)	0.655
TBIL (μmoI/L)	12.8 (9.78–16.43)	12.9 (9.90–16.48)	12.8 (9.68–16.20)	0.803
GGT (IU/L)	18.00 (13.00–25.00)	18.00 (13.00–25.00)	18.00 (13.00–25.00)	0.811
SCR (μmoI/L)	70.00 (57.85–79.90)	68.3 (57.85–79.40)	70.6 (57.48–80.93)	0.499
BUN (mmol/L)	4.40 (3.61–5.15)	4.40 (3.67–5.35)	4.30 (3.60–4.86)	0.486

*Data are median (interquartile range) or n (%)*.

*ALT, alanine aminotransferase; AST, aspartate aminotransferase; BUN, blood urea nitrogen; High-normal ALT, serum ALT between 35–40 IU/L in males and 25–40 IU/mL in females; GGT, γ-glutamyl transpeptidase; HBcAb, hepatitis B core antibody; HBeAg, hepatitis B envelop antigen; HBsAg, hepatitis B surface antigen; RBC, red blood cell; SCR, serum creatinine; S/CO, signal-to-cutoff ratio; TBIL, total bilirubin; PLT, platelet; WBC, white blood cell*.

### Clinical Assessment of Liver-Inflammation Grade

Patients with significant liver inflammation (G ≥ 2) were shown to have higher levels of ALT, AST, and GGT but lower white blood cell counts, platelet (PLT) counts, HBsAg levels, and HBV DNA levels (Table 2).

**Table 2:** Analysis of factors associated with the presence of significant inflammation (G ≥ 2).

**Variables**	**All**	**G < 2**	**G ≥ 2**	***P*-value**
Population	504	440	64	
Age (years)	38 (32–44)	37 (32–43.75)	40 (31–50)	0.089
Gender				0.366
Male (%)	294 (58.33)	260 (59.10)	34 (53.13)	
Female (%)	210 (41.67)	180 (40.90)	30 (46.87)	
High-normal ALT				0.003^*^
No (%)	381 (75.60)	342 (77.73)	39 (60.94)	
Yes (%)	123 (24.40)	98 (22.27)	25 (39.06)	
HBeAg status				0.838
Negative (%)	179 (35.52)	157 (35.68)	22 (34.38)	
Positive (%)	325 (64.48)	283 (64.32)	42 (65.62)	
Significant fibrosis				<0.001^*^
S < 2 (%)	376 (74.60)	354 (80.45)	22 (34.38)	
S ≥ 2 (%)	128 (25.40)	86 (19.55)	42 (65.62)	
ALT (IU/L)	25.00 (18.00–31.25)	24.00 (18.00–30.43)	30.00 (23.00–35.00)	<0.001^*^
AST (IU/L)	22.00 (18.70–26.00)	21.85 (18.00–25.00)	29.00 (23.00–33.75)	<0.001^*^
HBsAg (Log10 IU/mL)	3.81 (3.06–4.51)	3.82 (3.11–4.57)	3.66 (2.69–3.97)	0.008^*^
HBeAg (S/CO)	291.37 (0.21–1376.15)	805.10 (0.20–1389.99)	10.06 (0.39–832.02)	0.110
HBcAb (S/CO)	8.88 (0.01–11.41)	8.79 (0.01–11.39)	9.50 (4.28–11.95)	0.201
HBV DNA (Log10 IU/mL)	7.39 (3.90–8.28)	7.55 (3.86–8.34)	5.79 (4.27–7.76)	0.019^*^
WBC (10E9/L)	5.60 (4.67–6.66)	5.64 (4.7–6.75)	5.19 (4.48–6.11)	0.012^*^
RBC (10E12/L)	4.67 (4.30–5.04)	4.68 (4.3–5.06)	4.57 (4.32–5.00)	0.695
PLT (10E9/L)	192.00 (158.25–227.00)	197.00 (163.25–231.75)	157.50 (127.25–189.00)	<0.001^*^
TBIL (μmoI/L)	12.8 (9.78–16.43)	12.75 (9.8–15.77)	14.25 (9.45–18.10)	0.236
GGT (IU/L)	18.00 (13.00–25.00)	17.25 (13.00–24.00)	24.5 (18.25–33.50)	<0.001^*^
SCR (μmoI/L)	70.00 (57.85–79.90)	70.00 (57.4–79.95)	68.4 (64.50–78.00)	0.930
BUN (mmol/L)	4.40 (3.61–5.15)	4.41 (3.66–5.20)	3.9 (3.39–4.73)	0.174

*Data are median (interquartile range) or n (%)*.

*ALT, alanine aminotransferase; AST, aspartate aminotransferase; BUN, blood urea nitrogen; High-normal ALT, serum ALT between 35–40 IU/L in males and 25–40 IU/mL in females; GGT, γ-glutamyl transpeptidase; HBcAb, hepatitis B core antibody; HBeAg, hepatitis B envelop antigen; HBsAg, hepatitis B surface antigen; RBC, red blood cell; SCR, serum creatinine; S/CO, signal-to-cutoff Ratio; TBIL, total bilirubin; PLT, platelet; WBC, white blood cell*.

* *P < 0.05*.

### Construction of the Model and Nomogram

Through univariate logistic regression analysis, 9 variables were found to be significant at a level of *P* < 0.1 (Table 3). These candidate predictors were then evaluated by multivariate regression analysis. We performed stepwise regression using 3 methods (forward, backward, and both ways) to select the model with minimal Akaike's information criterion, all of which chose AST levels (IU/L), GGT levels (IU/L), PLT counts (^*^10e9/L), and HBsAg levels (log10 IU/mL) to construct the model, with variance inflation coefficients of 1.05, 1.06, 1.14, and 1.07, respectively. The coefficients suggested that these variables had no collinearity. The resultant model is as follows: Logit (Y) = −0.09 + 1.15 × AST + 0.07 × GGT −0.03 × PLT −0.65 × HBsAg.

**Table 3:** Univariate and multivariate regression analyses investigating the predictors for significant inflammation (training set).

	**Crude**		**Adjusted**	
	**OR (95% CI)**	***P*-value**	**OR (95% CI)**	***P*-value**
Age (years)	1.048 (1.008–1.090)	0.0173^*^	0.997 (0.95–1.046)	0.897
Gender (1 = male, 2 = female)	1.123 (0.537–2.318)	0.754		
ALT (IU/L)	1.077 (1.029–1.132)	0.002^**^	1.016 (0.935–1.106)	0.701
High-normal ALT (1 = yes, 0 = no)	2.078 (0.961–4.367)	0.056^*^	0.824 (0.226–2.991)	0.767
AST (IU/L)	1.152 (1.085–1.227)	<0.001^**^	1.157 (1.064–1.263)	<0.001^**^
GGT (IU/L)	1.063 (1.025–1.101)	<0.001^**^	1.067 (1.018–1.120)	0.007^**^
HBsAg (Log10 IU/mL)	0.598 (0.425–0.837)	0.003^**^	0.541 (0.325–0.889)	0.016^**^
HBeAg status (1 = positive, 0 = negative)	0.963 (0.456–2.141)	0.922		
HBcAb (S/CO)	1.004 (0.998–1.008)	0.115		
HBV DNA (Log10 IU/mL)	0.859 (0.735–1.002)	0.052^*^	0.974 (0.770–1.236)	0.824
WBC (10E9/L)	0.770 (0.583–0.988)	0.051^*^	0.927 (0.658–1.277)	0.653
RBC (10E12/L)	0.677 (0.298–1.429)	0.329		
PLT (10E9/L)	0.977 (0.967–0.986)	<0.001^**^	0.972 (0.959–0.985)	<0.001^**^

*ALT, alanine aminotransferase; AST, aspartate aminotransferase; CI, confidence interval; High-normal ALT, serum ALT between 35–40 IU/L in males and 25–40 IU/mL in females; GGT, γ-glutamyl transpeptidase; HBcAb, hepatitis B core antibody; HBeAg, hepatitis B envelop antigen; HBsAg, hepatitis B surface antigen; OR, odds ratio; RBC, red blood cell; S/CO, signal-to-cutoff Ratio; PLT, platelet; WBC, white blood cell*.

* *P < 0.1 and*

** *P < 0.05*.

**Figure 1:**
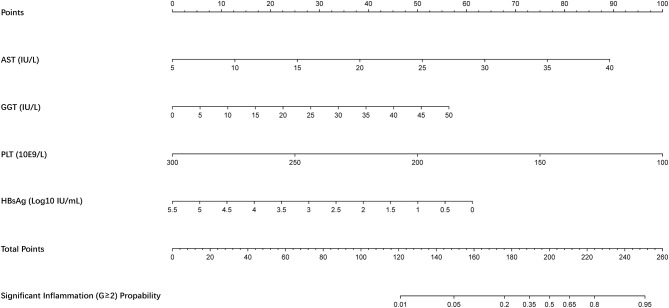
Nomogram for predicting significant liver inflammation in chronic hepatitis B patients with normal alanine aminotransferase. AST, aspartate aminotransferase; GGT, γ-glutamyl transpeptidase; HBsAg, hepatitis B surface antigen; PLT, platelet.

**Figure 2:**
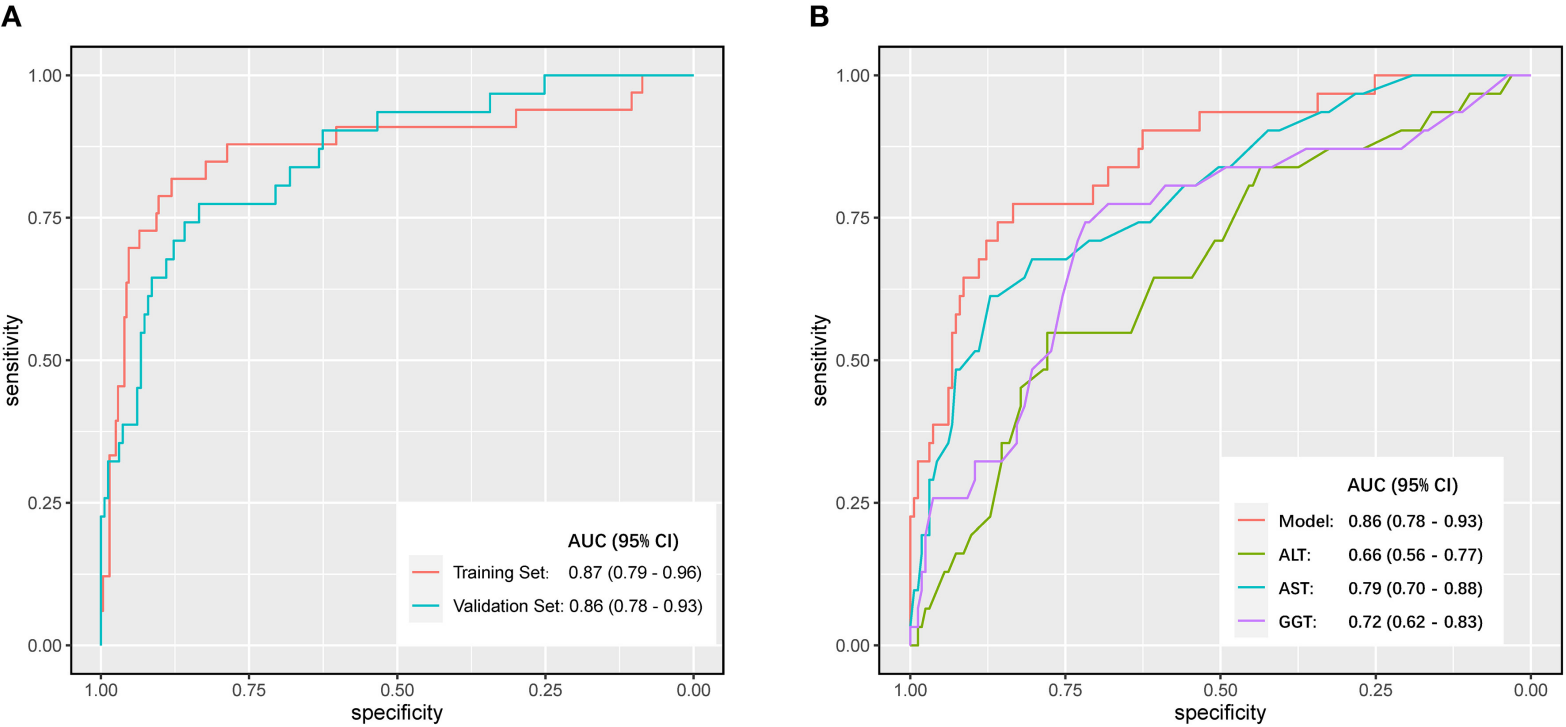
Discrimination performance was compared by Receiver Operating Characteristic (ROC) curves. **(A)** Comparison of the area under the curve (AUC) of the model in the training set and the validation set. **(B)** Comparisons of AUC between different independent predicting parameters (ALT, AST, GGT) and the model in the validation set.

**Figure 3:**
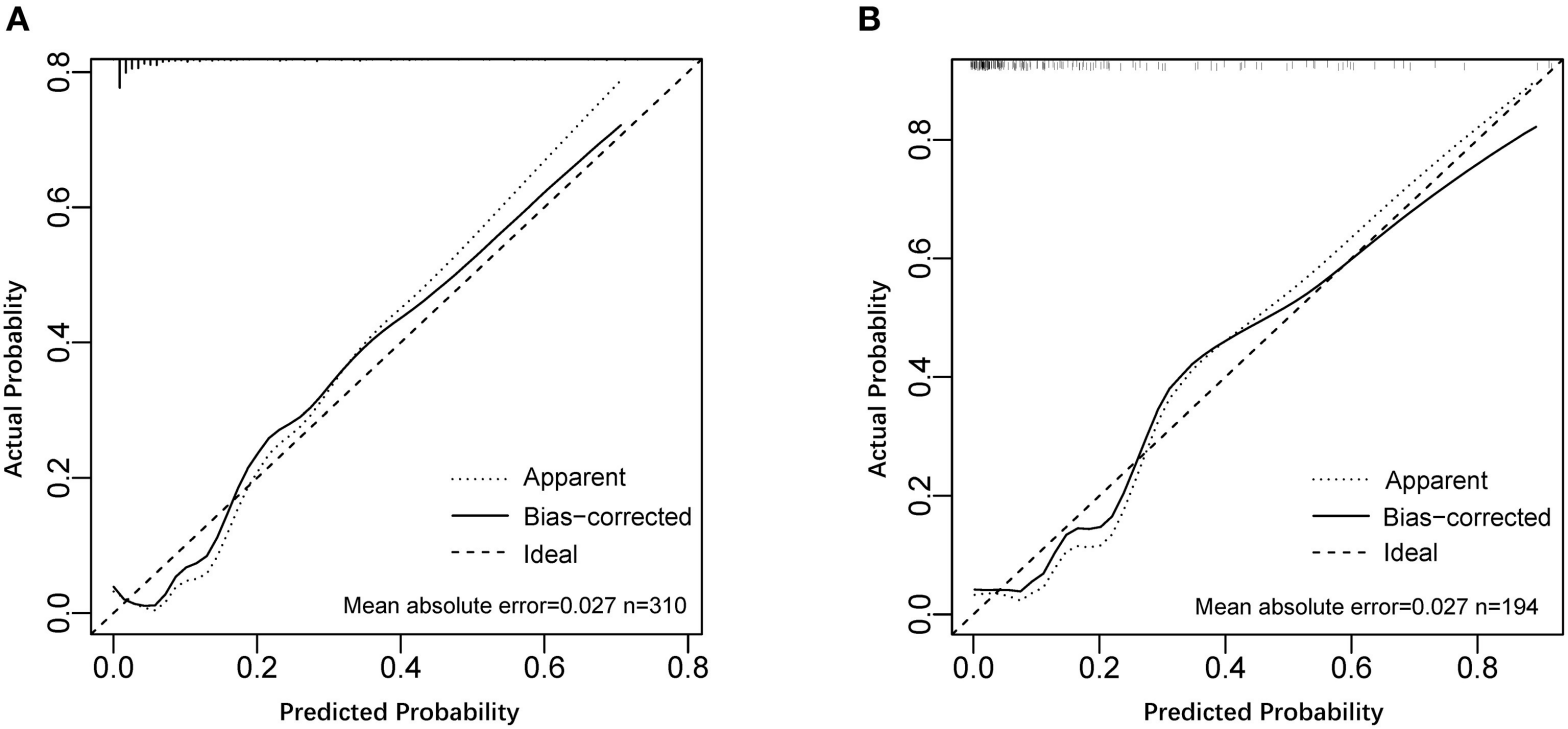
Calibration curves of the model for predicting significant liver inflammation. **(A)** Calibration curve of the model in the training set. **(B)** Calibration curve of the model in the validation set.

A nomogram incorporating these 4 predictors was constructed based on the model (Figure 1). The nomogram yielded an AUC of 0.87 (95% CI, 0.79–0.96) in the training set (Figure 2A). The calibration curve was close to the diagonal, showing good agreement between the predicted and observed probabilities (Figure 3A).

### Validation of the Model and Comparison Through Traditional Assessments

Good discrimination was obtained by validating the model using the validation set with an AUC of 0.86 (95% CI, 0.783–0.931) (Figure 2A), and a calibration curve indicated that the model had good calibration (Figure 3B).

We compared the model against independent predictors (ALT, AST, and GGT levels) for the diagnosis of significant liver inflammation in the validation set. The model presented a greater AUC than ALT (0.66; 95% CI, 0.56–0.77), GGT (0.72; 95% CI, 0.62–0.83), or AST (0.79; 95% CI, 0.70–0.88) levels alone (Figure 2B). The maximum AUC was at a model value cutoff = 0.12, with a specificity of 83.43%, sensitivity of 77.42%, positive likelihood ratio of 4.67, and negative likelihood ratio of 0.27.

### Clinical Significance of the Model

**Figure 4:**
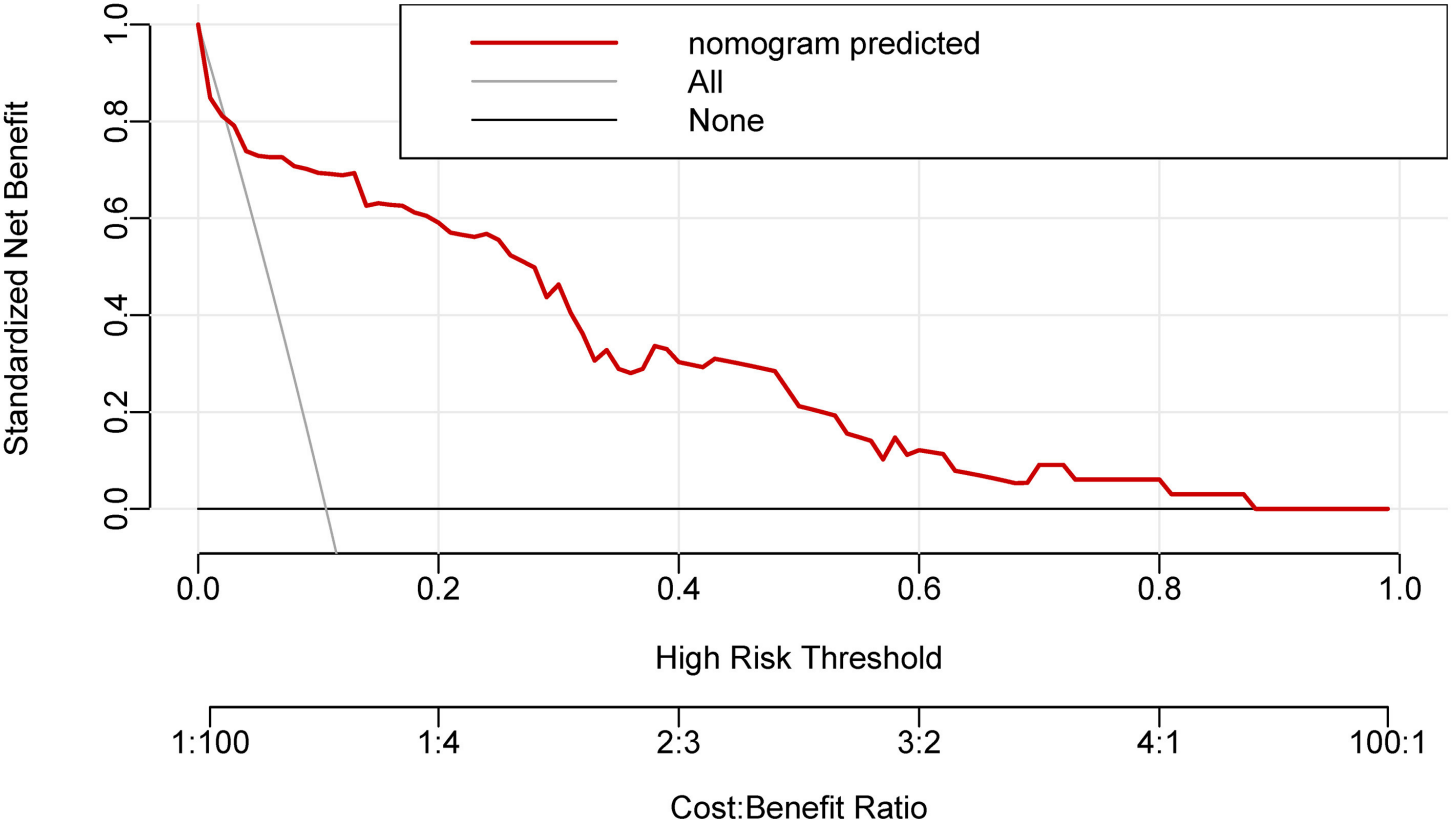
The decision curve analysis for the nomogram. The nomogram-based decision offered a net benefit over the “treat-all” or “treat-none” strategy at a threshold probability >3.0%.

The model offered a net benefit over the “treat-all” or “treat-none” strategy at a threshold probability >3.0% through DCA (Figure 4), which suggested that the model was clinically useful. For example, with a threshold probability of 20%, a nomogram-based prediction could provide an added net benefit of 25% compared to the treat-all or treat-none strategy.

## Discussion

The accumulation of extracellular matrix in the liver causes fibrosis, which is usually driven by hepatotoxic injuries. It regresses when the causative agent is withdrawn. Currently, hepatic fibrosis is treated in a passive manner involving control of liver inflammation through antiviral treatment (22). It is essential to detect inflammation and initiate antiviral treatment in a timely manner. While diverse serum indicators (e.g., type III precollagen, type IV collagen, laminin, and hyaluronidase levels), non-invasive examinations (e.g., TE), and models (e.g., the GPR, APRI, and FIB-4 index) are used for the diagnosis of liver fibrosis, few studies have been carried out on the diagnosis of significant liver inflammation in chronic liver disease. Xu et al. (23) reported that red blood cell distribution width levels correlate with liver fibrosis and inflammation in CHB patients. Wang et al. (24) also reported that red blood cell distribution width and globulin levels could predict liver inflammation and fibrosis. However, these studies focused on patients with normal or mildly elevated ALT levels. In this study, we focused on effectively diagnosing significant liver inflammation in a population with completely normal serum ALT levels.

According to current Chinese, APASL, EASL, and AASLD guidelines, liver biopsy is essential for evaluating the progression of HBV infection and defining the stage of the disease (4, 10–12). The AASLD recommends aggressive liver biopsy for those older than 40 with persistent ALT levels below 2 ULN, especially among those infected who are HBeAg (+) and have a DNA level >20,000 IU/mL or are HBeAg (−) and have a DNA level >2,000 IU/mL (10). In clinical practice, the diagnosis of liver histology is critical for determining whether to initiate antiviral therapy. The latest Chinese guidelines for CHB treatment recommend that patients with normal ALT levels who are DNA (+) start antiviral therapy if there is evidence of liver inflammation or fibrosis progression, regardless of viral load (10). This approach is more radical than that espoused by other guidelines, but it also reflects the importance of liver biopsy.

However, it is difficult to obtain permission for liver biopsy from individuals with normal serum liver enzyme levels. Furthermore, a consensus has developed that even among those with ALT levels that are persistently within the normal range, a proportion of patients with significant hepatocyte inflammation remain and are at risk for the development of cirrhosis and liver cancer (25, 26), for whom timely initiation of antiviral therapy is essential. If a patient with normal ALT levels does not consent to a liver biopsy, then TE can be used as an alternative to help doctors decide whether to start antiviral treatments. However, the principle of TE allows for the assessment of fibrosis, not hepatocellular inflammation. In individuals with hepatocellular inflammation, a high body mass index, or obesity, the results of TE may be confounded (21, 27, 28). Even if existing means are capable of accurately detecting liver fibrosis, it is still crucial to establish methods that accurately predict inflammation, given that inflammation is the initiating factor and primary cause of fibrosis.

Inflammation is commonly associated with all stages of liver diseases. Because it eventually drives the development of liver fibrosis, potential serum markers that predict liver fibrosis may therefore be applied to grade inflammatory activity. In this study, we observed that patients with significant liver inflammation had lower HBsAg and HBV DNA levels, lower platelet counts, and higher levels of serum liver enzymes, which is consistent with our previous study that reported a negative correlation between HBsAg titers and fibrosis stage in HBeAg (+) CHB patients (29). Although these patients, who did not receive antiviral therapy, had normal ALT levels, their lower HBsAg and DNA levels often suggested that they were far from being in the typical immune tolerance phase. In such cases, patients may experience hepatocyte inflammation due to the presence of immune-mediated viral clearance, and it would be beneficial to initiate antiviral therapy in a timely manner.

The cause of the low platelet counts in patients with significant inflammation is unknown; platelets have been reported to be crucial in the pathogenesis of acute and chronic liver disease associated with HBV infection by promoting the accumulation of virus-specific CD8+ T cells and non-specific inflammatory cells in the liver parenchyma (30). Whether exhaustion during chronic inflammation reduces the platelet count is open to debate. Due to the confounding presence of fibrosis and inflammation in those patients, significant inflammation possibly accompanies advanced fibrosis, which may also contribute to lower platelet levels. We will discuss this limitation shortly.

The normal ALT value range, which was initially explored in the construction of our model, remains to be determined. A lower ULN may help to delineate a larger population with mildly abnormal ALT levels, but it is worth investigating whether this change is useful for identifying patients with potential hepatocyte inflammation. In this study, we specifically designed a variable (named “high-normal ALT”) to observe the difference between the traditional ALT ULN and the adjusted ULN. After adjustment, it came to our attention that a decreased ALT ULN (from 40 to 35 IU/L in males and 25 IU/L in females) may not effectively further differentiate between individuals with mild and significant liver inflammation, despite higher ALT levels being found in the G ≥ 2 population. Subsequent studies are needed to identify a more reasonable ULN range in a larger sample.

The data showed that the new model had a good ability to discriminate between patients with moderate to high inflammation and those with mild or no inflammation. The novelty of this study is that it focused on the prediction of liver inflammation in a population with normal ALT levels. Each of the predictive variables is routinely tested, including HBsAg, PLT, and liver enzyme levels. Laboratory measurements of these variables are linear, have good reproducibility, and can be performed in most parts of China.

The study has the following limitations. The sample size was relatively small and inadequate for stratified analysis. Because the data were obtained from multiple clinical centers, it was difficult to find an independent, adequate data set for validation, so no external validation of the model was performed. Unlike other studies, we did not distinguish patients by HBeAg status, because according to the latest Chinese guidelines, for patients with normal ALT, the status of HBeAg (positive or negative) is no longer a decisive factor in deciding whether to initiate antiviral therapy. It was also noticed that HBeAg status was not found to be a predictor of liver inflammation in the initial stage of the univariate regression analysis. Another drawback of our study was that we did not include TE as a predictor variable for model building because TE is not yet widely available in China.

In this study, we selected patients with normal ALT, AST, and GGT levels. Given that this study relied on a retrospective data analysis, most patients did not experience long-term follow-up before undergoing liver biopsy, and because no repeated measurements of liver enzymes were performed, the lacking follow-up data caused uncertainties regarding the role of normal liver enzymes in these patients and also affected the diagnosis. It is possible that patients exhibited normal liver enzymes in a transient manner. It may have been more convincing if more liver function tests had been conducted prior and after the liver biopsy.

An important and unavoidable confounding factor is that patients presenting with liver inflammation often present comorbid fibrosis, which was also observed in the current study. Because we did not obtain data from enough patients with only liver inflammation and no fibrosis, which was due to the limitations of the data set, it was impossible to evaluate the degree of inflammation independently from fibrosis. Because the variables used in the current model (AST levels, GGT levels, and PLT counts) are also key variables in non-invasive diagnostic models of liver fibrosis (e.g., the APRI and GPR), we cannot rule out the possibility of the model being confounded by coexisting liver fibrosis.

## Conclusions

This study provides a non-invasive approach to diagnosing significant liver inflammation in people with chronic HBV infection and may help clinicians identify more hepatitis patients with fully normal ALT levels who potentially need antiviral treatment. The model can also be applied to identify high-risk individuals who need to undergo liver biopsies. It is believed that similar studies could be helpful in the diagnosis of other diseases that require liver biopsy (e.g., alcoholic liver disease, non-alcoholic fatty liver disease). It is also necessary to recognize that non-invasive diagnostic tools are currently a supplement, not a substitute for liver biopsy and TE. To distinguish those CHB patients with normal ALT, the HBV DNA, TE, and/or liver biopsy are still mandatory.

## Data Availability Statement

The raw data supporting the conclusions of this article will be made available by the authors, without undue reservation.

## Ethics Statement

The studies involving human participants were reviewed and approved by IRB of Dongzhimen Hospital affiliated to Beijing University of Chinese Medicine. The patients/participants provided their written informed consent to participate in this study. Written informed consent was obtained from the individual(s) for the publication of any potentially identifiable images or data included in this article.

## Author Contributions

XL did the statistical analysis and drafted the manuscript. YY conceived the study. XCh, GT, and YG were principal investigators in the medical centers, where the records were collected. SL, YX, HX, ZZ, LZ, ZL, PZ, JZ, HD, XCa, XZ, XS, and DZ helped to summarize the data. SL, LZ, and NZ polished the manuscript. All authors contributed to the article and approved the submitted version.

## Conflict of Interest

The authors declare that the research was conducted in the absence of any commercial or financial relationships that could be construed as a potential conflict of interest.

## Abbreviations

CHB: chronic hepatitis B
ALT: alanine aminotransferase
ROC: receiver-operating characteristic
DCA: decision curve analysis
AST: aspartate aminotransferase
GGT: γ-glutamyl transpeptidase
HBsAg: hepatitis B surface antigen
PLT: platelet
HBV: hepatitis B virus
AASLD: American Association for the Study of Liver Disease
APASL: Asian Pacific Association for the Study of the Liver
EASL: European Association for the Study of the Live
TE: transient elastography
APRI: aspartate aminotransferase-to-platelet ratio index
GPR: γ-glutamyl transpeptidase-to-platelet ratio
ULN: upper limit of normal.
